# Supplementary material for: Gene family expansion and functional diversification of chitinase and chitin synthase genes in Atlantic salmon (Salmo salar)
Source: G3 (Bethesda). 2023 Mar 27;13(6):jkad069. doi: 10.1093/g3journal/jkad069 (PMC10234404; doi:10.1093/g3journal/jkad069)
Supplement: jkad069_Supplementary_Data [file jkad069_supplementary_data.pdf]

File S1

Supplementary Table 1. Information about RNA-seq data used to compare tissue expression in different tissues across species.

Tissue	Species	Individuals	Project number	Database
Eye	Zebrafish	1	E-MTAB-8959	ArrayExpress
Gill	Zebrafish	1	E-MTAB-8959	ArrayExpress
Heart	Zebrafish	1	E-MTAB-8959	ArrayExpress
Liver	Zebrafish	4	E-MTAB-8959	ArrayExpress
Midgut	Zebrafish	1	E-MTAB-8959	ArrayExpress
Skin	Zebrafish	1	E-MTAB-8959	ArrayExpress
Eye	Northern pike	1	PRJNA221548	BioProject
Gill	Northern pike	1	PRJNA221548	BioProject
Head kidney	Northern pike	1	PRJNA221548	BioProject
Heart	Northern pike	1	PRJNA221548	BioProject
Liver	Northern pike	1	PRJNA221548	BioProject
Stomach	Northern pike	1	PRJNA221548	BioProject
Midgut	Northern pike	1	PRJNA221548	BioProject
Spleen	Northern pike	1	PRJNA221548	BioProject
Eye	Rainbow Trout	1	E-MTAB-8959	ArrayExpress
Gill	Rainbow Trout	1	E-MTAB-8959	ArrayExpress
Head kidney	Rainbow Trout	1	E-MTAB-8959	ArrayExpress
Heart	Rainbow Trout	1	E-MTAB-8959	ArrayExpress
Liver	Rainbow Trout	3	E-MTAB-8959	ArrayExpress
Pyloric caeca	Rainbow Trout	1	E-MTAB-8959	ArrayExpress
Midgut	Rainbow Trout	1	E-MTAB-8959	ArrayExpress
Spleen	Rainbow Trout	1	E-MTAB-8959	ArrayExpress
Skin	Rainbow Trout	1	E-MTAB-8959	ArrayExpress
Eye	Atlantic salmon	1	PRJNA72713	BioProject
Gill	Atlantic salmon	1	PRJNA72713	BioProject
Head kidney	Atlantic salmon	1	PRJNA72713	BioProject
Heart	Atlantic salmon	1	PRJNA72713	BioProject
Liver	Atlantic salmon	1	PRJNA72713	BioProject
Stomach	Atlantic salmon	15	PRJEB21981	European Nucleotide Archive
Pyloric caeca	Atlantic salmon	15	PRJEB21981	European Nucleotide Archive
Midgut	Atlantic salmon	167	PRJEB24480	European Nucleotide Archive
Spleen	Atlantic salmon	1	PRJNA72713	BioProject
Skin	Atlantic salmon	1	PRJNA72713	BioProject

Supplementary Table 2. Phylogeny-based annotation of glycoside hydrolase family 18- and chitin synthase genes and proteins in Atlantic salmon.

Clade	Gene name used in this paper	NCBI gene ID	Protein name used in this paper	RefSeq protein accession number
CHIA	*chia.3*	106565088	Chia.3	XP_013987246.1
	chia.4	106565087	Chia.4	XP_013987245.1
	chia.7	106572401	Chia.7	XP_014002002.1
CHIO I	*chia.2*	106565093	Chia.2	XP_013987254.1
	chia.9	106583514	Chia.9	XP_014023261.1
	chia.8	106577510	Chia.8	XP_014011074.1
	chia.6	106567267	Chia.6	XP_013991841.1
CHIO II	*chia.1*	106565309	Chia.1	XP_013987730.1
	chia.10	106583513	Chia.10	XP_014023260.1
CHIT1	*chia.5*	106567333	Chia.5	XP_013991941.1
CHID1	*chid1*	100194776	Chid1	NP_001133277.1
CTBS	*ctbs*	100195549	Ctbs	XP_014071095.1
CHS	*chs1a*	106589822	Chs1a	XP_014035674.1
	chs1b	106612488	Chs1b	XP_014069148.1
	chs2	106562657	Chs2	XP_013983103.1
	chs3	106570137	Chs3	XP_013997648.1

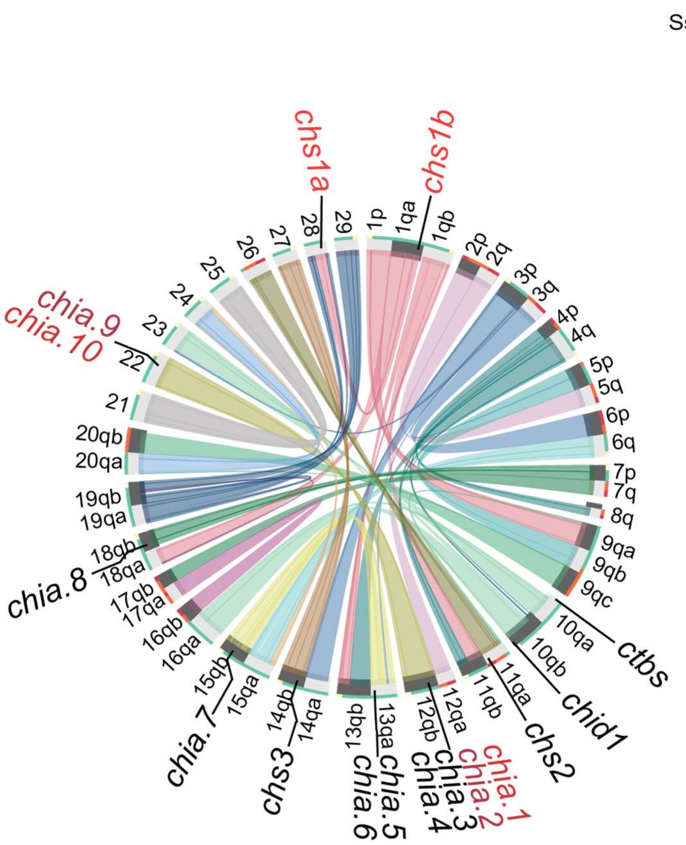

Supplementary Figure 1. Circos plot showing the chromosomal location of glycoside hydrolase family 18 and chitin synthase genes in Atlantic salmon. The blocks represent homologous regions in the genome with the 29 Atlantic salmon chromosomes, see (Lien *et al.* 2016) for more information. The genes marked in red (*chia.1* + *chia.10, chia.2* + *chia.9, chs1a* + *chs1b*) are located on homologous regions of different chromosomes and are hypothesized to result from the salmonid-specific whole-genome duplication event (Ss4R).

Supplementary Table 3. Results from the KEGG enrichment with pathways, number of genes, p-value, NCBI gene ID, and gene description.

KEGG Pathway	Genes	Genes in list	P-value	PathwayID	NCBI Gene ID	NCBI Gene description
Amino sugar and nucleotide sugar metabolism	84	6	8.79E-10	path:sasa00520	106560290	UDP-N-acetylhexosamine pyrophosphorylase-like
Amino sugar and nucleotide sugar metabolism	84	6	8.79E-10	path:sasa00520	106565093	Acidic mammalian chitinase-like (*chia.2*)
Amino sugar and nucleotide sugar metabolism	84	6	8.79E-10	path:sasa00520	106565309	Acidic mammalian chitinase-like (*chia.1*)
Amino sugar and nucleotide sugar metabolism	84	6	8.79E-10	path:sasa00520	106567267	Acidic mammalian chitinase-like (*chia.6*)

Amino sugar and nucleotide sugar metabolism	84	6	8.79E-10	path:sasa00520	106577510	Acidic mammalian chitinase-like (*chia.8*)
Amino sugar and nucleotide sugar metabolism	84	6	8.79E-10	path:sasa00520	106612488	Uncharacterized (*chs1b*)
Metabolic pathways	2355	13	2.43E-06	path:sasa01100	101448035	beta-carotene 15,15'-monooxygenase 1 like
Metabolic pathways	2355	13	2.43E-06	path:sasa01100	106560290	UDP-N-acetylhexosamine pyrophosphorylase-like
Metabolic pathways	2355	13	2.43E-06	path:sasa01100	106564978	beta-1,3-galactosyl-O-glycosyl-glycoprotein beta-1,6-N-acetylglucosaminyltransferase 3-like
Metabolic pathways	2355	13	2.43E-06	path:sasa01100	106565093	Acidic mammalian chitinase-like (*chia.2*)
Metabolic pathways	2355	13	2.43E-06	path:sasa01100	106565309	Acidic mammalian chitinase-like (*chia.1*)
Metabolic pathways	2355	13	2.43E-06	path:sasa01100	106567267	Acidic mammalian chitinase-like (*chia.6*)
Metabolic pathways	2355	13	2.43E-06	path:sasa01100	106572530	diacylglycerol kinase alpha-like
Metabolic pathways	2355	13	2.43E-06	path:sasa01100	106575817	acyl-CoA dehydrogenase, long chain
Metabolic pathways	2355	13	2.43E-06	path:sasa01100	106576053	aldose reductase-like
Metabolic pathways	2355	13	2.43E-06	path:sasa01100	106577510	Acidic mammalian chitinase-like (*chia.8*)
Metabolic pathways	2355	13	2.43E-06	path:sasa01100	106609639	ST8 alpha-N-acetyl-neuraminide alpha-2,8-sialyltransferase 1
Metabolic pathways	2355	13	2.43E-06	path:sasa01100	106609667	alpha-N-acetylneuraminide alpha-2,8-sialyltransferase-like
Metabolic pathways	2355	13	2.43E-06	path:sasa01100	106612488	Uncharacterized (*chs1b*)

Glycosphingolipid biosynthesis - ganglio series	29	2	0.00059208	path:sasa00604	106609639	ST8 alpha-N-acetyl-neuraminide alpha-2,8-sialyltransferase 1
Glycosphingolipid biosynthesis - ganglio series	29	2	0.00059208	path:sasa00604	106609667	alpha-N-acetylneuraminide alpha-2,8-sialyltransferase-like
Glycosphingolipid biosynthesis - globo and isoglobo series	30	2	0.00063388	path:sasa00603	106609639	ST8 alpha-N-acetyl-neuraminide alpha-2,8-sialyltransferase 1
Glycosphingolipid biosynthesis - globo and isoglobo series	30	2	0.00063388	path:sasa00603	106609667	alpha-N-acetylneuraminide alpha-2,8-sialyltransferase-like
Glycosphingolipid biosynthesis - lacto and neolacto series	46	2	0.00148954	path:sasa00601	106609639	ST8 alpha-N-acetyl-neuraminide alpha-2,8-sialyltransferase 1
Glycosphingolipid biosynthesis - lacto and neolacto series	46	2	0.00148954	path:sasa00601	106609667	alpha-N-acetylneuraminide alpha-2,8-sialyltransferase-like
Retinol metabolism	74	2	0.00380361	path:sasa00830	100196171	putative all-trans-retinol 13,14-reductase
Retinol metabolism	74	2	0.00380361	path:sasa00830	101448035	beta-carotene 15,15'-monooxygenase 1 like
Glycerolipid metabolism	100	2	0.00683169	path:sasa00561	106572530	diacylglycerol kinase alpha-like
Glycerolipid metabolism	100	2	0.00683169	path:sasa00561	106576053	aldose reductase-like

References

Lien S, Koop BF, Sandve SR, Miller JR, Kent MP, Nome T, Hvidsten TR, Leong JS, Minkley DR, Zimin A, et al. 2016. The Atlantic salmon genome provides insights into rediploidization. Nature. 533(7602):200–205. doi:10.1038/nature17164.
